# Challenges in managing urinary tract infection and the potential of a point-of-care test guided care in primary care: an international qualitative study

**DOI:** 10.3399/bjgpopen18X101630

**Published:** 2019-04-03

**Authors:** Lucy Brookes-Howell, Emma Thomas-Jones, Janine Bates, Marie-Jet Bekkers, Curt Brugman, Elinor Coulman, Nick Francis, Khurram Hashmi, Kerenza Hood, Nigel Kirby, Carl Llor, Paul Little, Michael Moore, Anna Moragas, Kate Rumsby, Theo Verheij, Christopher Butler

**Affiliations:** 1 Research Fellow (Qualitative), Centre for Trials Research, College of Biomedical and Life Sciences, Cardiff University, Cardiff, UK; 2 Research Fellow, Centre for Trials Research, College of Biomedical and Life Sciences, Cardiff University, Cardiff, UK; 3 Research Associate, Centre for Trials Research, College of Biomedical and Life Sciences, Cardiff University, Cardiff, UK; 4 Research Associate, Centre for Trials Research, College of Biomedical and Life Sciences, Cardiff University, Cardiff, UK; 5 Project Manager, Julius Center for Health Sciences and Primary Care, UMC Utrecht, Utrecht, The Netherlands; 6 Research Associate, Centre for Trials Research, College of Biomedical and Life Sciences, Cardiff University, Cardiff, UK; 7 Professor, Division of Population Medicine, Cardiff University, Cardiff, UK; 8 GP Academic Fellow, Division of Population Medicine, School of Medicine, Cardiff University, Cardiff, UK; 9 Professor, Centre for Trials Research, College of Biomedical and Life Sciences, Cardiff University, Cardiff, UK; 10 Senior Data Manager, Centre for Trials Research, College of Biomedical and Life Sciences, Cardiff University, Cardiff, UK; 11 GP and Researcher, Division of Population Medicine, School of Medicine, Cardiff University, Cardiff, UK; 12 Professor, Primary Care & Population Sciences, University of Southampton, Southampton, UK; 13 Professor, Primary Care & Population Sciences, University of Southampton, Southampton, UK; 14 Project Manager, University Institute in Primary Care Research Jordi Gol, Via Roma Health Centre, Barcelona, Spain; 15 GP and Associate Professor, University Rovira i Virgili. Primary Healthcare Centre Jaume I, Tarragona, Spain; 16 Study Manager, Primary Care & Population Sciences, University of Southampton, Southampton, UK; 17 Professor, Julius Center for Health Sciences and Primary Care, UMC Utrecht, Utrecht, The Netherlands; 18 Professor of Primary Care, Nuffield Department of Primary Care Health Sciences, University of Oxford, Oxford, UK

**Keywords:** general practice, point-of-care testing, urinary tract infections, diagnosis, qualitative research, antibiotics

## Abstract

**Background:**

Little is known about clinicians’ experiences of using a point-of-care test (POCT) to inform management of urinary tract infection (UTI) in general practice.

**Aim:**

To explore experiences of using the Flexicult test to inform management of UTI and views on requirements for an optimal POCT to inform successful implementation.

**Design & setting:**

Telephone interviews with 35 primary care clinicians and healthcare professionals in Wales, England, Spain, and the Netherlands, who had participated in a trial of the Flexicult POCT for UTI based on urine culture.

**Method:**

Thematic analysis of semi-structured interviews.

**Results:**

Most primary care clinicians interviewed agreed on the need for a POCT in UTI management, and that the Flexicult POCT delivered quicker results than laboratory results used in usual care, reassured patients, boosted their confidence in decision-making, and reminded them about antibiotic stewardship. However, clinicians also reported difficulties in interpreting results, limitations on when the Flexicult could be used, and concerns that testing all patients would strain care delivery and prolong patient discomfort when delaying decisions until a non-rapid POCT result was available. An optimal POCT would produce more rapid results, and be reliable and easy to use. Uptake into routine care would be enhanced by: clear guidance on which patients should be tested; training for interpreting ‘grey area’ results; reiterating that even ‘straightforward’ cases might be better managed with a test; clear messages about stopping unnecessary antibiotics versus completing a course; and better self-management strategies to accompany implementation of delayed, or non-prescription of, antibiotics.

**Conclusion:**

Primary care clinicians believe that POCT tests could play a useful role in the management of UTI and gave clear recommendations for successful implementation.

## How this fits in

Symptoms attributable to UTI are usually treated empirically with antibiotics despite evidence that many episodes do not benefit from antibiotics, or are caused by organisms resistant to conventional empirical antibiotics. Current tools to aid management of UTI either require waiting several days to receive results (laboratory-based urine culture) or provide results with sub-optimal sensitivity and specificity (urine dipsticks). POCTs of urine culture (such as Flexicult) can indicate organism quantification and sensitivity within 24 hours, and have the potential to influence antibiotic prescribing decisions. This qualitative study examining primary care clinicians’ perspectives on the use of POCTs found that they perceived a need for POCTs, and made a series of clear recommendations for successful uptake and implementation.

## Introduction

Antibiotics play a key role in improving health and wellbeing. However, their use has increased exponentially, leading to the selection and spread of antibiotic resistance. Antimicrobial resistance is one of the most serious threats to public health.^1^ Reductions in antibiotic use is associated with reductions, or at least limiting increases, in antibiotic-resistant UTIs.^2^ Resistant UTIs are symptomatic for longer and are more expensive to treat in primary care.^3,4^ Appropriate use of existing antibiotics is therefore a European and international priority.

GPs prescribe the majority of antibiotics used in health care.^5^ Therefore, improving the quality of antibiotic prescribing decisions in primary care can make an important contribution to reducing antimicrobial resistance.^5^ Of all community antibiotic prescriptions, 15% are issued for uncomplicated UTIs.^6^ Not all women with symptoms associated with a UTI have a bacterial infection, and non-bacterial bladder and urethral irritation can cause symptoms that are assumed to originate from bacterial UTI, and are thus inappropriately treated with antibiotics.^7^ Diagnostic accuracy of clinical assessment^8,9^ and optimal management of uncomplicated UTI is unclear.^10^ Most women presenting with UTI symptoms are prescribed an antibiotic.^11^ However, empirical antibiotics are poorly targeted. Current strategies to predict bacteriological UTI need refining, and there is an urgent need to support GPs in deciding when to prescribe antibiotics and, when required, the most appropriate choice of antibiotic.

A recent UK government report, commissioned to address the growing global problem of drug-resistant infections, created a ‘*diagnostic wish list*’ which included the '*rapid AMR* [antimicrobial resistance] *assessment directly on urine samples for patients with suspected UTI*'.^12^ The final report^13^ states that the use of rapid diagnostics is key. A recent review of horizon scan reports to identify evidence gaps in POCT evaluation found that assessment of the broader impact of diagnostic technologies (for example, acceptability, and societal and organisational consequences) are often overlooked.^14^ There is a need to understand clinicians’ reasoning and their requirements for specific tests, in order to target interventions so that they are useful in a real-life setting and lead to behaviour change.

POC testing for UTI was carried out in the primary care (POETIC) trial.^15^ This was a pragmatic, parallel two-arm, individually randomised controlled trial comparing the effects and costs of the Flexicult test-guided diagnostic and treatment strategy for symptoms of uncomplicated UTI in adult women on the overall appropriateness of antibiotic use when compared to standard care. Flexicult is an optimised POCT for quantification and determining sensitivities of UTI used in primary care, with results available within 24 hours

In the POETIC trial, only a third of participants had a laboratory confirmed UTI, and 58.4% were assessed as being prescribed antibiotics inappropriately.^16^ The main driver of inappropriate usage was use of antibiotics in women whose laboratory urine culture results did not confirm UTI (49.5%). Use of Flexicult POCT urine culture was found to result in fewer women being prescribed antibiotics at the initial consultation (82.4% versus 88.4%), but by day 3, more participants in the control arm were appropriately taking (or not taking) antibiotics (44.1% versus 39.3%).

Participating clinicians generally prescribed antibiotics for patients in the POCT arm without waiting for the Flexicult results, and seldom withdrew antibiotic treatment when the test indicated no UTI.

Therefore, this qualitative study aimed to gain a deep understanding of clinicians’ views about management and diagnosis of UTI in adult women in general, as well as their experiences of using the Flexicult to inform their management decision. More specifically, it aimed to explore the challenges clinicians face in diagnosing and managing UTI, and the extent to which a POCT (such as Flexicult) can be used in an individual clinician's everyday practice setting.

## Method

Semi-structured telephone interviews were conducted with 35 primary care clinicians in research networks in four European countries (Wales, England, Spain, and the Netherlands), all of whom participated in the POETIC trial. Each network aimed to recruit 10 clinicians. Clinicians in the Spanish network were purposefully sampled to include high, middle, and low recruiters into the trial to ensure they had a varied range of experience of using the POCT. A total population sample was used in the other three networks, where there were fewer clinicians to select from, and all clinicians had to be contacted to obtain a sufficient sample size.

Recruitment took place between July–September 2015. Verbal informed consent was taken and audio-recorded over the telephone by the local researchers.

The interview topic guide was developed by the team's qualitative research fellow, in collaboration with interviewers. Telephone interviews were conducted locally by research staff in Spain (a GP and local POETIC Trial researcher), in the Netherlands (a project officer and local POETIC trial researcher), and in England and Wales (one GP and clinical research fellow, and one qualitative research associate). All received detailed face-to-face training in qualitative interviewing techniques, which included theoretical background and the need to reflect on their own professional role in asking and interpreting interview questions, as well as role play and discussion. Interviews were carried out in the clinician’s chosen language and audio-recorded. Interviews were semi-structured and consisted of three broad topic sections (typical management for women with suspected UTI, opinions on the Flexicult POCT, and participation in the trial), as shown in Box 1. All interviews were transcribed and translated into English for analysis.

Box 1. **POETIC clinician interview topic guide**

**Table:** 

**Typical management for adult women with suspected UTI** Could you tell me about your usual approach to diagnosis of suspected UTI in adult women? Could you now describe your usual approach to management of suspected UTI in women?
**The POCT: Flexicult** Can you describe how you integrated using the Flexicult into your consultation? What didn’t work well about using the Flexicult? What worked well about using the Flexicult? Could Flexicult help you decide whether or not to prescribe an antibiotic for uncomplicated UTI? During your time in the trial, do you feel the way you managed patients assigned to standard care altered as a result of you participating in this research? Do you feel that there is a need for a POCT in managing UTI? We are beginning our preliminary analysis on the use of the Flexicult. We haven’t finished analysis yet, but its looking like antibiotics were sometimes changed, but not so often stopped. Why do you think this was? Do you think antibiotic resistance in UTI is an important problem in your practice?
**The trial** Did your expectations of what would be required of you match your actual experience of being in the trial? Were there any aspects of the trial that you thought worked well? Were there any aspects of the trial that you thought did not work well? How could the trial have been done better, from your perspective?

POCT = point of care test. UTI = urinary tract infection

**Figure 1.**
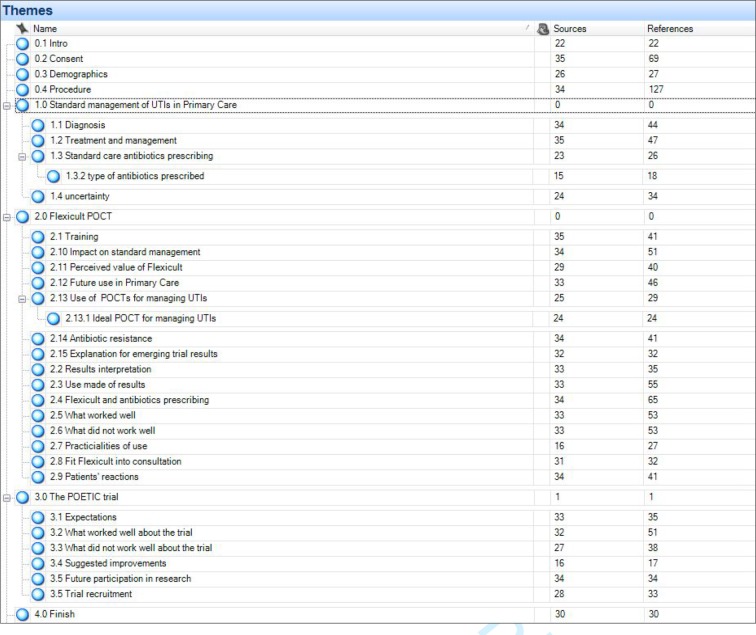
Thematic framework developed to analyse interview data

Transcripts were analysed using a thematic analytic method. This involved data familiarisation, generating initial codes, searching, reviewing, and defining themes.^17^ A thematic framework was developed. A deductive approach was used to incorporate the research objectives and topic guide, and an inductive approach was used to ensure that new themes, which had not been pre-empted and initiated from the clinicians themselves, were identified in the data. Figure 1 shows the full list of themes and sub-themes used for analysis. The thematic framework was applied to data using qualitative software NVivo (version 10). Developments in the analytic process were recorded through researcher memos and version control of the codebook. Of the transcripts, 20% were double-coded, reaching 96% agreement, and coding discrepancies were discussed and consensus reached.

## Results

Interviews were conducted with 35 primary care clinicians and healthcare professionals in Wales (*n* = 8), England (*n* = 8), Spain (*n* = 10), and the Netherlands (*n* = 9). More female clinicians were interviewed than males (27 female versus 8 male). In total, 19 GPs and 16 other healthcare professionals (nurses *n* = 10, practice nurses *n* = 3, nurse practitioner *n* = 1, research nurse/research manager *n * = 1, healthcare assistant *n *= 1) were interviewed. The number of years since clinicians qualified ranged from 4–40 years (those most recently qualified clinicians in the Netherlands [mean 13 years], and those qualified longest in England [mean 27.5 years]).

Representative quotes are provided, followed by network code and clinician’s study number (see Box 2).

Box 2. **Illustrative data quotes**

**Table:** 

Extract	Clinician quote
**Challenges of diagnosing and managing suspected UTIs in everyday practice**	
1	*'If they* [the patients] *are er fairly sure they have an uncomplicated UTI, i.e., no systemic features, er dysuria frequency and urgency, or at least two out of those three um, they’re happy with the diagnosis, they’re over eighteen, they’re not pregnant, um, and they don’t have any significant allergies or anything, we would usually prescribe them a three day course of trimethoprim over the phone. *[I: 'Okay and, er, how confident do you feel in making the diagnosis of UTI?] *'Reasonably.'* (England, GP, 3)
2	*'Sometimes it can be extremely difficult… Initial diagnosis can be, well, um, thrush in particular. Um, sometimes you can be, um, very confident but there are, there are cases where it can be quite, quite difficult and in those cases we would send the specimen for MSU.'* (Wales, Practice Nurse, 7)
3	*'If the nitrite does not show anything and we use a culture and sometimes it is contaminated urine, so then it looks like there are bacteria on it while this is sometimes not the case. In most instances this is the case, but sometimes it may give an incorrect result, that some mixed flora is shown.*' (the Netherlands, Nurse, 7)
**Using Flexicult POCT to manage suspected UTI in a real practice setting**	
4	*'I absolutely feel it* [the Flexicult] *would be added value to a GP practice because you are able to give more targeted and specific treatment… because you know straight away which antibiotics you should give, so I think that in the future those dipslides will become a thing of the past, that this Flexicult will become a nice innovation.'* (the Netherlands, Nurse, 4)
5	*'I am now more aware that not everything should be identified as an urinary tract infection* […] *The only problem is that our only tool is the Labstix* […] *Maybe this is just temporary and afterwards we will revert to our usual habits, and you don't want to miss any infections.'* (Spain, GP, 8)
6	*'I think we are probably more inclined with a lot of patients to send the samples and contact the patient if it comes back with an, with an infection rather than you know, just giving in to the patient I suppose when they’re sitting in front of you.'* (Wales, Nurse, 5)
7	*'That would be quite exciting* [I: mmhm] *just to see something in front of me because obviously I don’t work in a lab, I haven’t been in a lab for years* [I: mmhm] *but it was something that I could see* [I: mmhm] *working or not working.* […] *even the receptionist* [I: mmhm] "*What are you doing?"* [I: mmhm] *"What are you growing?"* [I: mmhm] *so they were involved in the study.'* (England, Practice Nurse, 5)
**Using Flexicult POCT to manage suspected UTI in a real practice setting: disadvantages**	
8	*'It’s all about time* [I: yes, yes yes] *so um, I know, at the end of the day, I wouldn’t, I certainly wouldn’t want to be doing er Flexicult on every single patient* […] *if this became routine to us* [I: mmhm] *well, we would be doing four times more, er, Flexicult than we did* [in the trial] [I: right] *and that would certainly need another, another member of staff, person to perform those tests really.'* (Wales, GP, 1)
9	[On whether Flexicult might be used in the future in routine care] *'I wonder if it will actually happen, because they are expensive, they are more expensive than dipslides.'* (the Netherlands, Nurse, 4)
10	*'Obviously if it was Friday, it was difficult because we weren’t going to be there and they did quite quickly overcook. I don’t know how well, erm, thermostatically regulated our oven was but, erm, if you left it much beyond the 24 hours because you forgot or a bit, er, too busy it would already begin to take an overall brown appearance.'* (England, Nurse, 2)
11	*'By the time we finished we felt a lot more confident and actually felt we were doing it properly, er, and reading it off. I think I got, er, the doctors to look at the first one or two with me* […] *I think it became more useful probably just as we got more used to it.'* (England, Nurse, 2)
**Using Flexicult POCT to manage suspected UTI in a real practice setting: managing patient expectations**	
12	*'At first I found it a bit difficult, I mean, yes, it will give you useful information in 24 hours but at the precise moment when you diagnose, then, and actually, it didn’t change my approach that much.'* (Spain, GP, 6)
13	*'With a type of acute urinary tract infection where people have a lot of symptoms,* […] *people simply have a fever, or something like that, well then you are not going to wait. And some people, it is also a kind of service orientation, if people have a lot of symptoms and say they cannot cope, then you just cannot wait 24 hours.'* (the Netherlands, Nurse, 3)
14	*'They liked it because they felt we were keeping an eye on them.'* (Spain, GP, 9)
15	*'I think ladies are willing to, um, wait, uh, would be willing to wait more for a prescription if they knew that they were going to be getting some results in say 24 hours, as opposed to having to wait three or four days for a lab result.'* (England, Practice Nurse, 6)
**Impact of Flexicult on antibiotic prescribing in a real practice setting**	
16	*'It was very positive and useful, because you would obtain the results in a very short period of time, so you would be able to contact the patient if the antibiotic you had prescribed wasn’t the right one. It is a technique which could be very useful in primary care.'* (Spain, GP, 1)
17	[On the need for fast diagnosis systems for uncomplicated patients] *'I believe we don’t* […] *Actually, we don’t even use urine strips. Because it is actually very easy, with empirical treatment, in Spain at least, a monodose and that’s it.'* (Spain, GP, 9)
18	*'I don’t feel it was a hugely time-consuming exercise, performing that, and if the patient, if a sample was requested it could be done, it didn’t have to be done there and then, it could be done later on* [I: mmhm] *later on in the morning.'* (Wales, Research Nurse, 8)
**Requirements of an optimal POCT**	
19	*' … having it as a tool when in doubt, or in cases where, we sometimes speculate with treatment, to cover, don’t we? Because, in these cases, yes it would help. When you think "I wouldn’t prescribe it, but as I might have to wait a week for the urine culture, and it is a long time, then I prescribe it". If we had the Flexicult, in these cases, then maybe yes.'* (Spain, GP, 2)
20	[On whether a POCT is useful in managing UTI] *'It, as I say, has not altered my, um, my prescribing um performance* [I: yeah, mmhm] *uhm, I don’t think it showed that I was missing* [signs] [I: mmhm] *I don’t think it showed that I was prescribing, er, antibiotics inappropriately, I think it, you know, my clinical judgment, I think, er, was fairly consistent with the results of the, uhm, cultures.'* (Wales, GP, 1)
21	*'Turns a straightforward consultation into something that takes a long time* [I: right] *and then you’ve got to follow it up again the next day, so we just don’t have the time to do that.'* (England, GP, 4)
22	*'It might seem quite easy, it’s easy to see a UTI, and just give people antibiotics and send them on their way* [I: mmhm] *but actually properly unpicking it and doing it properly longer term may save us a lot more in the future. So I reckon as a point of care test it is needed, it’s helpful.'* (Wales, GP, 2)

MSU = midstream urine. POCT = point of care test. UTI = urinary tract infection.

### Challenges of diagnosing and managing suspected UTI in everyday practice

All clinicians described their usual practice of diagnosing suspected UTI as combining patient symptoms and history information. Clinicians in all networks talked about the use of dipsticks to support their clinical diagnosis. However, accounts of when and why the dipstick was used varied and included convenience (if they '*have one handy*' [Spain, GP, 10]) and practice borne over time ('*my old habits die hard*' [England, Nurse, 2]). In the Netherlands, dipslides^18^ were also used frequently. In England, Wales, and the Netherlands, clinicians reported asking patients to complete a checklist prior to, or instead of, face-to-face consultation. GPs, citing their experience, tended to express more confidence than nurses in being able to diagnose UTIs accurately. GPs in the Spanish network quantified their confidence level at 70–80%, while those in England, Wales, and The Netherlands were generally more guarded, saying they were '*reasonably*' confident in their diagnosis (Box 2, extract 1). Clinicians openly acknowledged the potential difficulties in getting the diagnosis right. In all networks, midstream urine samples (MSU) were sent to the microbiology laboratory when the clinicians were in any doubt, or if symptoms had not resolved after a first short course of antibiotics (Box 2, extract 2). Clinicians described challenges in diagnosis, centring around factors such as vague symptoms, contaminated MSU samples, and/or mixed growth (Box 2, extract 3). Treatment decisions were usually made after face-to-face contact and dipstick testing. However, if the UTI seemed straightforward and was not recurrent, decisions were also based on telephone consultations.

### Using a POCT (Flexicult) to manage suspected UTI in a real practice setting

#### Advantages: confidence boosting, speedy results, awareness raising, and interesting technology

Clinicians expressed increased confidence in diagnosing UTI, as the Flexicult complemented their assessment of symptomatic patients. The test both confirmed the presence of UTI and indicated which antibiotic was appropriate to prescribe, offering more targeted treatment within 24 hours instead of the 3–4 days it usually took to obtain laboratory results (Box 2, extract 4). Clinicians felt that this opportunity for faster results, compared to an MSU, was one of the test’s greatest advantages.

In addition to increasing confidence and producing faster results than laboratory tests, some clinicians described the test as having an impact on awareness raising for themselves as health professionals. They felt that the test had a positive impact on standard care during the trial as it acted as a reminder that potential UTI symptoms may indicate different underlying conditions. Furthermore, many reported that this awareness stayed with them in the post-trial period (Box 2, extract 5). Additionally, some clinicians felt that using Flexicult brought home the issue of prudent antibiotic prescribing and antibiotic resistance (Box 2, extract 6).

A number of clinicians engaged in the opportunity to use novel technology, with some describing how interesting it was to see science in action. Nurse practitioners, in particular, described taking a personal interest in studying the test results. Some reported that they had gone to read the plates on their off days, and one described carrying around the incubator between practice locations on her moped. Where interpretation of results became a joint effort between practice staff, this helped to create a positive work atmosphere (Box 2, extract 7).

### Disadvantages and problems: strain on resources and interpretation of plates

In spite of its generally positive reception, clinicians showed a keen awareness of the various tensions between the need to provide fast and reliable diagnoses and treatment, and the limitations inherent in increasingly overburdened healthcare systems. Singled out in this respect were staffing issues, with little or no spare capacity to prepare, cultivate, read, and follow up on the results of the test. Clinicians felt that this problem would be compounded if Flexicult were to be used for all suspected UTI cases, rather than just a limited number of trial recruits (Box 2, extract 8). Practicality and cost were also mentioned, as clinicians were concerned about the short shelf life of the plates and potential expense associated with maintaining regular stock, suggesting that integrating the test into routine workflow might be problematic (Box 2, extract 9). In addition, standard weekend practice closures meant that Flexicult use had to be in many practices as ‘*a Monday to Thursday thing*’ (Wales, GP, 1), as no one was available to record test results over the weekend (Box 2, extract 10).

Ease of interpretation was an issue for some clinicians, as some experienced difficulties in interpreting the test (Box 2, extract 11). They indicated that actual plates did not conform neatly to the examples presented during training and handbook pictures.

### The use of a POCT and managing patient expectations

Some clinicians expressed concern about waiting to obtain test results (Box 2, extract 12). They felt that some patients would be in too much discomfort to wait for a result and would prefer immediate antibiotics (Box 2, extract 13). However, others reported that patients felt reassured, as if their situation was being monitored, implying increased level of care and treatment (Box 2, extract 14). Some felt that the test could be used to make delayed scripts more acceptable to patients (Box 2, extract 15).

### Impact of Flexicult on antibiotic prescribing in a real practice setting

At the time of interview, the emerging trial results indicated antibiotics were sometimes changed to an appropriate antibiotic if there was resistance (Box 2, extract 16), but were frequently not stopped, even after a test indicated no UTI. Interviewees were asked to reflect on this and provide a possible explanation based on their own experience. The majority of responders surmised that, as treatment for UTIs tends to be for 3–5 days, patients may have already taken most of their medication before they could be contacted, and that finishing a course of antibiotics might be seen as the more important health message. In Spain, a single ‘monodose’ may be prescribed, meaning that by the time the Flexicult result was available, patients would have completed the antibiotic regime (Box 2, extract 17). Others indicated that the trial results did not reflect their own practice, as they *did* stop unnecessary antibiotics or inform patients not to use deferred scripts.

The test was perceived as helpful in prescribing for people whose MSU results came back as mixed growth. It was also deemed potentially more important in older people with recurrent UTIs rather than uncomplicated UTI.

Overall reactions to the Flexicult test were positive, with a minority of health professionals saying that they saw little value in the test. Most thought that problems associated with the test (such as limited staff capacity and cost) were offset by the perceived advantages of the test, or the difficulties were deemed to be manageable (Box 2, extract 18). However, for situations where the practitioner felt quite confident that they were making the right diagnosis, the test was judged to offer little added value to current practice.

The perceived impact of Flexicult use on antibiotics prescribing appears evenly divided between ‘no change’ and ‘more awareness and therefore more cautious prescribing habits’. For some responders, Flexicult allowed delayed scripts to be used, which patients could pick up after results were available. For others, antibiotics were prescribed as per local protocols but, importantly, if the results indicated that a change in the prescription was necessary, then patients would be contacted.

### Requirements of an optimal POCT

When considering the perceived value of the Flexicult test in future practice, the majority of clinicians felt that it had value for UTI management. Other clinicians were mostly fairly positive about the value of the test but either felt it would only be needed for complicated cases (Box 2, extract 19), or that it would not be viable in its current form due to staffing issues or cost. The two remaining clinicians categorically expressed that they would not use Flexicult in future UTI management due to the time it took to do the test, or that the test had not changed their treatment decisions (Box 2, extract 20). Clinicians had varying views about the usefulness of the test in various scenarios. One clinician in England felt that the test would add extra time onto a *'straightforward consultation'* (Box 2, extract 21), and therefore was not particularly useful. However, a clinician in Wales felt that using the test to unpick an '*easy*' case of UTI, might save time in the future by getting the diagnosis and management right quicker, and limiting the need for patients to re-consult (Box 2, extract 22). Some clinicians felt the test would be most useful for specific patient groups including those with '*innocuous symptoms*' (Wales, GP, 2), older patients, young patients, and males.

When describing the ‘ideal’ test, the key component seemed to be fast results (instant or same-day), with ease of use, and accuracy and reliability mentioned far less. Many, particularly clinicians in the Netherlands cited Flexicult as the ideal test, but some felt that it would be better if the results were available quicker. One suggested a quicker version of the test, which used a midstream urine sample to show strong, mild, or moderate pathogen immediately, the same stick then being placed in an incubator to read the next morning .

## Discussion

### Summary

Overall reactions to the urine culture POCT were positive. The perceived impact of Flexicult use on antibiotic prescribing appears evenly divided between ‘no change’ and ‘more awareness and therefore more cautious prescribing habits’. The randomised controlled trial (RCT) associated with this study showed that antibiotics were sometimes changed but were frequently not stopped, even after the test indicated no UTI. This qualitative study showed that most clinicians felt that this may be because finishing a course of antibiotics might be seen to be the more important health message.

Clinicians overwhelmingly felt that a POCT for UTI management would be useful. When describing the ‘ideal’ test, the key component seemed to be fast results, while ease of use and accuracy and reliability were mentioned far less. Many described the Flexicult POCT as the ideal test but some felt that it would be better if it gave faster results.

### Strengths and limitations

Qualitative interviews were used to capture the experiences of actual trial implementers on the use of the Flexicult test in the POETIC trial, rather than asking clinicians to reflect hypothetically on POCT use in UTI management.

Views on UTI management and POCT use were captured across different European countries.

Participating clinicians were affiliated to a research network so may not have been representative of all clinicians in their country.

Qualitative interviews gather reports of behaviour and attitude rather than actual behaviour, but by allowing clinicians to introduce and elaborate on themes spontaneously, the authors were able to gain an impression of the themes that held most prominence for the clinicians themselves.

### Comparison with existing literature

This interview study and the results of the RCT^16^ showed that, while many clinicians reported the need for a POCT for patients with UTI symptoms, they also expressed a relatively high degree of confidence in diagnosis of UTI based on clinical practice. This discrepancy has important implications for the successful uptake of a POCT in practice; as Cals *et al* explained, POCTs only offer added value when the clinician is uncertain about the diagnosis.^19^

The clinicians in this study expressed concerns about Flexicult similar to those described by Wood *et al* in relation to a POCT for lower respiratory tract infection.^21^ In particular, they identified time to result, simplicity of interpreting test result, and increased workload involved in carrying out the test as potential problems. Clinicians considering hypothetical use of a POCT in Wood *et al* most commonly expressed concern about test accuracy, but this concern was not highlighted by the clinicians interviewed in the present study, where the POCT was actually used in practice.^20^

Some clinicians expressed concern about interpreting results, in that the culture growths did not look like the examples in training. Hullegie *et al* analysed the results of the associated RCT and confirmed major discrepancies between clinicians’ interpretations of the POCT versus laboratory staff interpretations of photographs and laboratory culture results. They warn that photographs of the urine culture plates should not be considered as a reliable diagnostic device.^21^

This study found that the waiting time to the test result was important to clinicians. This corresponds with previous work identifying criteria to evaluate the use of a potential POCT.^19^ In considering how fast a POCT needs to be, the authors of that study stated that the minimum requirement is that the result must be available within the timeframe in which a decision must be made.^19^ Therefore, for the Flexicult POCT to be truly useful, the present authors suggest clinicians need to re-define the decision-making period for UTI management to include the 24 hour period following the consultation, rather than limit it to the timeframe of the consultation itself.

Previous research^16,22^ has shown that antibiotic prescribing for UTI is not improved by clinicians knowing organism sensitivity, and that test specificity and being able to confirm a diagnosis of UTI is clinically more important. This study showed that clinicians did not frequently stop patients taking antibiotics, even after the POCT results indicated no UTI. Therefore, ways to encourage clinicians to use the test to inform patients to stop taking antibiotics are needed.

### Implications for research and practice

In order to understand whether clinicians would use a POCT in practice, it was necessary to understand whether they felt that diagnosing and managing UTI was a challenge. If clinicians perceive some UTI cases as a straightforward consultation, then the uptake of a test is likely to be less successful. GPs tended to express more confidence than nurses in being able to diagnose correctly, although clinicians openly acknowledged the potential difficulties in getting the diagnosis right.

The POETIC trial found 58.4% of women may have been prescribed antibiotics inappropriately, suggesting that there is a discrepancy between the confidence in diagnosis expressed by clinicians and actual practice. Critically, the clinicians in the RCT tended to diagnose UTI if there was some growth on the plate, when the laboratory considered that there was insufficient growth to meet the threshold for a laboratory diagnosis of a UTI.^16^ This relates to Finucane’s call to rethink the management of the urinary microbiome, as urine is not sterile and the urinary tract hosts diverse microbiota. Therefore, a definition of UTI based on bacteriuria being present may result in inappropriate antibiotic treatment which results in no change.^7^ It is possible that, to a clinician faced with a symptomatic woman and some growth in the urine, the imperative to stop antibiotics is not strong in that context; for example, the technology alone is not sufficient to convince the clinician to stop antibiotics. This would imply that a test is needed that is more closely aligned with patient symptoms and predicting benefit from antibiotics, than one which predicts a laboratory threshold for organism quantification to meet a threshold for UTI diagnosis.

It was found that some clinicians felt that the test had limited value in uncomplicated cases and situations where they felt quite confident that they were making the right diagnosis. Therefore, there is a need to emphasise to clinicians that research shows that UTI is difficult to diagnose effectively without the aid of test results; even in situations when the clinician thinks that a case is straightforward, they may be incorrect in their diagnosis and therefore a POCT may be beneficial in assisting diagnosis and guiding treatment decisions. Future training should also be modified to include ‘grey areas’ in interpretation of the Flexicult results.

It was also found that clinicians perceived the Flexicult as being more helpful for older patients, and that they had concerns with using the test in a non-trial setting, when the test could be used for all patients. Future work needs to consider whether a POCT would be used for all patients or selected cases.

Regarding the value of knowing organism sensitivities at the point of care, very few UTIs caused by resistant organisms were found, so the value of knowing sensitivities at the point of care for uncomplicated UTIs is probably not great.

There needs to be clinician education for using test results to stop antibiotics once prescribed, as well as changes in antibiotic type. In the wider context of doctor–patient communication, a clinician asking a patient to stop taking antibiotics they had previously prescribed may be seen as a face-threatening act,^23^ thus making the clinician appear uncertain to the patient, and having a detrimental effect on the doctor-patient relationship with regards to the patient’s trust in their clinician’s skills. Clinicians should be provided with communicative strategies to manage perceived uncertainty with patients without appear to lack in confidence or expertise. Some clinicians reported that patients felt reassured with the idea of the accurate results that Flexicult offered 24 hours later, so stressing the need for clinicians to delay decisions in order to use their expertise to interpret test results would be key. Education with clinicians needs to clarify the most important health message to pass on to patients, whether it is stopping unnecessary antibiotic treatment, or finishing the antibiotic course once started.

Finally, it is important to recognise the social context of the clinician faced with a symptomatic patient, and their ability to address patient preference for antibiotics, and/or desire to alleviate the patient’s discomfort. Strategies are needed to encourage clinicians to advise patients with UTI symptoms to delay starting antibiotics until a POCT result is known. One way to do this would be to provide clinicians with an alternative to offer symptomatic patients if they are delaying prescribing or stopping antibiotics. Giving clinicians a package of self-management strategies to ‘prescribe’ to patients instead of antibiotics may make it easier and more socially acceptable for clinicians to delay, or not prescribe, antibiotics to women experiencing symptomatic discomfort.

## References

[1] World Health Organization (2015). Global action plan on antimicrobial resistance. http://www.who.int/antimicrobial-resistance/publications/global-action-plan/en/.

[2] Butler CC, Dunstan F, Heginbothom M (2007). Containing antibiotic resistance: decreased antibiotic-resistant coliform urinary tract infections with reduction in antibiotic prescribing by general practices. Br J Gen Pract.

[3] Alam MF, Cohen D, Butler C (2009). The additional costs of antibiotics and re-consultations for antibiotic-resistant Escherichia coli urinary tract infections managed in general practice. Int J Antimicrob Agents.

[4] Butler CC, Hillier S, Roberts Z (2006). Antibiotic-resistant infections in primary care are symptomatic for longer and increase workload: outcomes for patients with *E. coli* UTIs. Br J Gen Pract.

[5] Costelloe C, Metcalfe C, Lovering A (2010). Effect of antibiotic prescribing in primary care on antimicrobial resistance in individual patients: systematic review and meta-analysis. BMJ.

[6] Mazulli T (2002). Resistance trends in urinary tract pathogens and impact on management. J Urol.

[7] Finucane TE (2017). 'Urinary tract infection' and the microbiome. Am J Med.

[8] Little P, Turner S, Rumsby K (2006). Developing clinical rules to predict urinary tract infection in primary care settings: sensitivity and specificity of near patient tests (dipsticks) and clinical scores. Br J Gen Pract.

[9] Bent S, Nallamothu BK, Simel DL (2002). Does this woman have an acute uncomplicated urinary tract infection?. JAMA.

[10] Car J (2006). Urinary tract infections in women: diagnosis and management in primary care. BMJ.

[11] Little P, Merriman R, Turner S (2010). Presentation, pattern, and natural course of severe symptoms, and role of antibiotics and antibiotic resistance among patients presenting with suspected uncomplicated urinary tract infection in primary care: observational study. BMJ.

[12] O'Neill J (2015). Rapid diagnostics: stopping unnecessary use of antibiotics. Review on antimicrobial resistance.

[13] O’Neill J (2016). Tackling drug-resistant infections globally: final report and recommendations. Review on antimicrobial resistance.

[14] Verbakel JY, Turner PJ, Thompson MJ (2017). Common evidence gaps in point-of-care diagnostic test evaluation: a review of horizon scan reports. BMJ Open.

[15] Bates J, Thomas-Jones E, Pickles T (2014). Point of care testing for urinary tract infection in primary care (POETIC): protocol for a randomised controlled trial of the clinical and cost effectiveness of FLEXICULT^™^ informed management of uncomplicated UTI in primary care. BMC Family Pract.

[16] Butler CC, Francis NA, Thomas-Jones E (2018). Point-of-care urine culture for managing urinary tract infection in primary care: a randomised controlled trial of clinical and cost-effectiveness. Br J Gen Pract.

[17] Braun V, Clarke V (2006). Using thematic analysis in psychology. Qualitative Research in Psychology.

[18] Winkens R, Nelissen-Arets H, Stobberingh E (2003). Validity of the urine dipslide under daily practice conditions. Fam Pract.

[19] Cals J, van Weert H (2013). Point-of-care tests in general practice: hope or hype?. Eur J Gen Pract.

[20] Wood F, Brookes-Howell L, Hood K (2011). A multi-country qualitative study of clinicians' and patients' views on point of care tests for lower respiratory tract infection. Fam Pract.

[21] Hullegie S, Wootton M, Verheij TJM (2017). Clinicians' interpretations of point of care urine culture versus laboratory culture results: analysis from the four-country POETIC trial of diagnosis of uncomplicated urinary tract infection in primary care. Fam Pract.

[22] Holm A, Cordoba G, Møller Sørensen T (2017). Effect of point-of-care susceptibility testing in general practice on appropriate prescription of antibiotics for patients with uncomplicated urinary tract infection: a diagnostic randomised controlled trial. BMJ Open.

[23] Brown P, Levinson SC (1987). Politeness: some universals in language usage.

